# Empathic Disequilibrium in Autistic Traits and CU Traits: Investigating Empathy Imbalance in Children

**DOI:** 10.1007/s10802-025-01405-y

**Published:** 2026-02-06

**Authors:** Giorgos Georgiou, Ido Shalev, Kostas A. Fanti, Florina Uzefovsky

**Affiliations:** 1 Department of Social and Behavioral Sciences, European University Cyprus, P.O. Box 22006, 1516 Nicosia, CY Cyprus; 2 MRC Cognition and Brain Sciences Unit, University of Cambridge, Cambridge, CB2 1TN UK; 3 University of Cyprus, Nicosia, Cyprus; 4 Ben Gurion University, Be’er Sheva, Israel

**Keywords:** CU Traits, Autistic Traits, Empathic Disequilibrium, Affective Empathy, Cognitive Empathy

## Abstract

The current study investigated the relationship between CU traits, autistic traits, and empathic disequilibrium, which reflects the imbalance between cognitive empathy (CE) and affective empathy (AE). Based on previous findings with adults, we hypothesized that children with elevated CU or autistic traits exhibit either AE or CE dominance in their empathy profiles. A total of 163 children aged 4 to 10 years (Mage = 7.30) participated in the study and were evaluated using reliable parent-report instruments. Polynomial regression with response surface analysis (PRRSA) was used to analyse the relationships between empathic disequilibrium and trait scores, while adjusting for age, sex, and overall empathy levels. The results indicated a notable association between AE dominance and autistic traits, reinforcing previous findings that suggest a different developmental profile of children with autistic characteristics. Conversely, lower levels of empathic disequilibrium (both AE and CE dominance) were linked to CU traits. CU traits were also related to a general reduction in empathy across both empathy types, indicating a broader empathic deficit. Additionally, age showed a positive correlation with both CU and autistic traits, while no gender differences were observed for either trait. These findings imply that empathic disequilibrium could serve as a valuable framework for understanding the emotional profiles of children with autistic traits and CU traits.

**Supplementary Information:**

The online version contains supplementary material available at 10.1007/s10802-025-01405-y.

## Introduction

Callous Unemotional (CU) traits encompass a collection of characteristics indicative of a callous disregard for others, a lack of remorse or empathic concern, superficial or deficient emotional responses, and an absence of concern regarding performance (Frick & White, 2008). Autistic traits, on the other hand, pertain to a spectrum of characteristics that exist below the diagnostic threshold for Autism Spectrum Disorder (henceforth referred to as ‘autism’, a term more widely endorsed by members of the autistic community; (Kenny et al., 2016), with difficulties in understanding the mental states and perspectives of others constituting some of the core features of these traits (Baron-Cohen, 2000; Constantino & Todd, 2003; Hill & Frith, 2003). Despite differences, both traits are associated with deficits in emotional processing, particularly empathy (Georgiou et al., 2019a; Hill & Frith, 2003; Jones et al., 2010).

Empathy is defined as an individual’s capacity to perceive and comprehend the actual or anticipated emotional states of others and to feel for the other, which is essential for effective social interactions (Hoffman et al., 2009). It is a multidimensional construct comprising two subcomponents: cognitive empathy (CE), which involves recognizing and evaluating others' emotions, and affective empathy (AE), which entails feeling for the other (Baron-Cohen & Wheelwright, 2004; Blair, 2005; Decety & Jackson, 2004). According to the Empathy Imbalance Theory (Smith, 2006), individuals with higher autistic traits may exhibit deficits in cognitive empathy but possess intact affective empathy. However, some empirical studies have demonstrated typical cognitive or even heightened affective empathy (Gillespie-Lynch et al., 2017; Lombardo et al., 2016). Regarding those with high CU traits, it is suggested that they often lack AE, but their CE remains unaffected. Nevertheless, literature presents contradictory findings, with some studies indicating deficits in AE (e.g., Anastassiou-Hadjicharalambous & Warden, 2008; Blair, 2005; Jones et al., 2010) and some showing deficits specific to CE (e.g., Chabrol et al., 2011; Georgiou et al., 2019b). This suggests that the notion of ‘deficits’ in empathy may not accurately reflect an individual's empathic capacities. Alternatively, examining the interplay between the two dimensions of empathy has been suggested to provide a more accurate description of how empathy relates to its outcomes. Consequently, in the present study, we explore the relationship between CU and autistic traits and empathic disequilibrium, which pertains to the tendency to experience a disparity in AE compared to CE (or vice versa) (Shalev et al., 2023).

### Empathy, Autistic and CU Traits

Research indicates that one key trait differentiating children with CU traits from other children exhibiting antisocial behavior is their lack of empathy (for a review, see Fanti, 2018). Consequently, the diagnosis of Conduct Disorder (CD) in the Diagnostic and Statistical Manual of Mental Disorders-5 (American Psychiatric Association & Association, 2013) includes a specifier called “Limited Prosocial Emotion” (LPE), which describes those individuals who engage in antisocial behavior and simultaneously show low levels of empathy, guilt, and remorse. In his review, Blair (2013) suggested that the empathy deficits seen in individuals with high CU traits stem from genetic and prenatal influences that disrupt normal neural development, particularly affecting amygdala, striatal, and ventromedial prefrontal cortex activity. Typically, individuals with CU traits and CD diagnosis are thought to have deficits primarily in AE, but not in CE. However, as stated before, research presents conflicting findings, with some studies identifying deficits in AE, while others point to deficits in CE alone (e.g., Anastassiou-Hadjicharalambous & Warden, 2008; Chabrol et al., 2011; Georgiou et al., 2019b; Jones et al., 2010).

In contrast, autism has often been associated with the opposite pattern, with intact AE, and reduced CE, with accounts suggesting that the characteristics of children diagnosed with autism represent a mirror image of those seen in children with high CU traits (Baron-Cohen, 2013). Dziobek and her colleagues (2008) suggested that autistic individuals experience impairments in CE while maintaining intact AE. Similarly, (Broekhof et al., 2015) revealed that children diagnosed with autism struggle more than typically developing children in grasping others’ desires and beliefs. Beyond clinical populations, individuals may exhibit characteristics associated with autism without necessarily meeting the diagnostic criteria (e.g., Constantino & Todd, 2003; Piven et al., 1997 for a comprehensive review, see Hill & Frith, 2003), often referred to as “autistic traits”. Aligned with this framework, several findings indicate that autistic traits are distinctly linked to lower CE (e.g., Dziobek et al., 2008; Lockwood et al., 2013). However, neurophysiological research indicates that autistic traits might also correlate with deficits in AE. Specifically, Klapwijk et al. (2016) discovered that boys exhibiting elevated levels of CU and autistic traits experience reduced left amygdala activity when exposed to angry and fearful faces, compared to typically developing peers, primarily linked to AE deficits. Similar findings were revealed for adults (Mazza et al., 2014) and children (Butean et al., 2014), raising questions about this inconsistency.

### Εmpathic Disequilibrium

This inconsistency raises inquiries, which give rise to the concept of empathic disequilibrium. Specifically, the concept of empathic disequilibrium is based on the observation that inconsistent findings regarding empathy and its relation to several psychological outcomes, such as autism and CU traits, can be attributed to our separate focus on AE and CE (Shalev & Uzefovsky, 2020; Shalev et al., 2023). Empathic disequilibrium proposes that it is not the individual levels of AE and CE that are significant, but rather the imbalance between them that may clarify the difficulties individuals experience. It is well established that AE and CE possess distinct underlying neurobiological, developmental, and genetic trajectories (De Waal & Preston, 2017; Shamay-Tsoory, 2008). From a developmental perspective, AE emerges early in human development (Davidov et al., 2021; Thompson, 1987), whereas mature CE is recognized later in development and is associated with Theory of Mind (TOM) (Carlson et al., 2013; Dorris et al., 2022; Uzefovsky & Knafo-Noam, 2016). Nonetheless, significant evidence suggests that AE and CE are interconnected, influencing one another. In their review, Torre and Lieberman (2018) highlight the connection between brain regions associated with CE and AE, suggesting a dynamic causal model that appears to promote balance and self-regulation. This indicates an interdependent relationship in which our understanding and perspective on various matters and the emotions of others shape our emotional experiences. (e.g., Lei et al., 2019; Shalev et al., 2023). We acknowledge that other models of autism propose that empathy-related differences and difficulties arise from the interactions between autistic and non-autistic individuals, as highlighted in Milton’s Double Empathy Problem (Milton et al., 2012). However, in this study, we focus on empathic disequilibrium as an interpersonal feature – specifically the imbalance between AE and CE within the individual – as our framework for understanding empathy in relation to autistic traits.

To date, empathic disequilibrium has predominantly been utilized in studies concerning autism (Moseley et al., 2024; Shalev & Uzefovsky, 2020; Shalev et al., 2022, 2023). Reassessing previous research on empathy in autism through the lens of empathic disequilibrium has yielded a novel framework, as empathic disequilibrium is predictive of autism diagnosis and autistic traits beyond mere empathy. Specifically, results revealed an empathic imbalance favouring AE (AE-dominance), which predicts autism diagnosis and social domains of autistic traits (e.g., Shalev & Uzefovsky, 2020; Shalev et al., 2022, 2023). These links were thought to reflect heightened emotional sensitivity, where feelings of others are intensely felt but relatively less easily understood, leading to an overwhelming experience in interpersonal interactions.

To the best of our knowledge, there exists a scarcity of studies examining this concept in relation to psychopathic traits, particularly concerning CU traits. Shalev and colleagues (2023) investigated empathic disequilibrium across various psychological conditions, including psychopathic traits, and uncovered an association with CE-dominance, but only after adjusting for heightened emotional reactivity. This points to a nuanced profile that warrants further investigation, in which some individuals with psychopathic traits are characterised by CE-dominance, indicating an ability to comprehend the emotions of others, alongside a comparatively diminished emotional resonance with these individuals’ feelings in adult individuals. Considering that both traits are evident from an early age, this prompts whether we can find similar outcomes in younger individuals. Moreover, while the clinical features of autism and CD are considered distinct and warrant further investigation, autistic and CU traits tend to be positively associated in the general population (D. Murphy et al., 2024), and the unique contribution of each to empathic disequilibrium has not been examined to date.

### Empathy in Childhood

Research indicates that typically developing children acquire CE and AE very early on, even during the first year of life. As children's behavior repertoire widens, children start to react to the other's distress with comforting behaviors (Knafo et al., 2009; Young et al., 1999; Zahn-Waxler et al., 1992). Despite this event, the majority of studies investigating the relation of autism, autistic and CU traits and empathy involve older children and adolescents (for autism and autistic traits see (Shalev et al., 2025). Therefore, a limited number of studies demonstrate that children with high levels of CU and autistic traits show alterations in empathy compared to typically developing children (Dadds et al., 2009; Lombardo et al., 2007). An event that underscores the necessity of addressing differences in empathy in early development is the finding that high levels of CU were associated with deficits in both empathy components in early childhood (Dadds et al., 2009). However, the developmental relationship between these components has only recently begun to receive attention. In the only longitudinal study to date investigating empathic disequilibrium in children aged 3 to 12, those with higher CU traits at the final time point remained in a state of AE-dominance for a longer period and, on average, did not reach equilibrium during the study period (Shalev et al., 2025). In contrast, children with fewer CU traits exhibited a period of AE-dominance that appears typical in development and gradually declined with age, a pattern not observed in children with high CU traits.

This observation may imply that the profile examined in younger children and adolescents does not necessarily reflect the same characteristics of these individuals in later developmental stages. Moreover, the links between empathic disequilibrium and autistic traits during development have not yet been investigated. Consequently, examination of empathic disequilibrium among children with elevated CU or autistic traits necessitates further investigation during development. This is essential for identifying the underlying mechanisms associated with these distinct psychological conditions.

### Current Study

The limited studies examining empathic disequilibrium in relation to CU and autistic traits underscore the necessity of this study. These factors, along with the need to conduct more studies involving younger children, shape the primary objective of this research: to investigate the association between CU, autistic traits, and empathic disequilibrium in children. In this study, a sample of children aged 4 to 10 years was used. Given the inconsistencies in previous findings on empathy components in individuals with autism (Broekhof et al., 2015; Klapwijk et al., 2016) and CU traits (Anastassiou-Hadjicharalambous & Warden, 2008; Georgiou, et al., 2019b), this study does not assume a specific directional relationship. Rather, it aims to examine the potential associatins between both trait dimensions and empathic disequilibrium, including the possibility that neither trait is significantly related to this imbalance.

The last aim of this study was to investigate the influences of age and sex on empathic disequilibrium. As previously indicated, young children exhibiting high CU traits may not have fully developed their capacity to recognise others' emotional states (i.e., cognitive empathy), whereas older children may demonstrate enhancements in this dimension of empathy (Dadds et al., 2009; Mullins-Nelson et al., 2006). Consequently, it is plausible to observe a significant main effect of age, even among children between the ages of 4 and 10. In terms of sex, while empathic disequilibrium has been consistently shown to be sex-sensitive in adulthood (Shalev & Uzefovsky, 2020; Shalev et al., 2022, 2023), a previous study found no difference in empathic disequilibrium during childhood (Shalev et al., 2025), suggesting that such differences may emerge later in development. While this warrants careful examination and replication, based on the current evidence we expect that no differences will be observed between boys and girls.

## Methods

### Participants

Details and further demographics of this dataset are provided in Georgiou et al., (2019a, 2019b). A subset of 163 parent–child dyads was selected from a larger screening sample of 1,652 preschool and primary school children living in the Republic of Cyprus. The selected subset was comprised of children randomly drawn from two groups: children with low empathy (below 1 SD; *N* = 78) and children with average to high empathy (within −1SD to + 1SD; *N* = 85). Despite this classification, children’s cognitive and affective empathy were approximately normally distributed with a mean slightly above 1 (Table 1). This is consistent with the typical range of empathy reported in Dadds et al. (2008). The mean age of the sample was 7.30 (± 1.43), ranging from 4 to 10 years, with slightly more boys than girls (55.78% boys). 8% of the children were in kindergarten, and 80% attended grades 1–3 of elementary school. Most responders were mothers (89.8%). Informed consent was obtained from all parents included in the study. The study followed the ethical standards of relevant institutional and national committees of the Centre of Educational Research and Assessment of Cyprus, Pedagogical Institute, Ministry of Education and the Culture and Cyprus National Bioethics Committee, and complied with the Helsinki Declaration.

**Table 1:** Descriptive statistics and missing data

Variable	Mean (SD)	% Missing data
Child’s age	7.30 (1.43)	11.59%
Child’s gender	55.78% boys	10.37%
SRS	41.77 (21.31)	21.95%
ICU	0.91 (0.41)	9.76%
GEM*		
Cognitive empathy	1.21 (1.40)	8.54%
Emotional empathy	1.13 (1.20)	7.32%

*Note*. Mean and standard deviation for key and demographic variables are reported. SRS – Social Responsiveness Scale; ICU – Inventory of Callous-Unemotional; GEM – Griffith Empathy Measure. * The mean item score for each subscale prior to centering is reported to provide meaningful means

### Power Analysis

A-priori power analysis was performed using 5,000 Monte-Carlo simulations in the *simsem* package v.5–16 (Pornprasertmanit et al., 2021). Based on previous findings linking empathic disequilibrium and autistic traits and psychopathic tendencies in adults (Shalev & Uzefovsky, 2020; Shalev et al., 2022), we expected to find a small to moderate effect size (*r* = 0.25). The generation of the population model was based on previous findings of the relationship between cognitive and emotional empathy (Dadds et al., 2008; Shalev & Uzefovsky, 2020). The simulation accounted for covariates assuming moderate-high correlation between autistic traits and callous-unemotional traits as reported using similar measures (Lineback et al., 2024). Based on these assumptions, the analysis estimated that our sample would achieve moderate power (1 – β = 0.70) to detect effects at a significance level of *α* < 0.05.

### Missing Data Handling

The overall percentage of missing data was 11.59%. Table 1 displays the percentage of missing data and descriptive statistics of each key variable. Insignificant Little’s test indicated that data were likely missing completely at random (χ^2^(32)** = **43.9, *p* = 0.08), minimizing the risk of bias using appropriate data completion methods. Since variables were approximately randomly distributed, full information maximum-likelihood was employed as the method of choice (Dong & Peng, 2013), implicated in the *lavaan* package v.6–18 (Rosseel, 2012).

### Empathy

Empathy was measured using the 23-item parent-report Griffith Empathy Measure (GEM; Dadds et al., 2008). Except for an overall empathy score, GEM captures the two subcomponents of empathy: cognitive and affective. In the current study only the two subcomponents were used and not the overall score. The cognitive empathy subscale is composed of 6 items (e.g., “My child has trouble understanding other people’s feelings”), and the affective empathy subscale of 9 items (e.g., “Seeing another child sad makes my child feel sad”). GEM is rated on a 9-point Likert scale (rating from − 4 = strongly disagree to 4 = strongly agree). Total scores range from − 92 to 92, with higher scores indicating higher levels of empathy. Prior studies have demonstrated good test–retest reliability of scores over 1 week (r > 0.89) and 6 month intervals (r > 0.69), good internal consistencies, a stable factor structure across age and sex groups, inter-parental agreement (r > 0.47), and good convergence with child reports (r = 0.41) (Dadds et al. 2008). Both affective (α = 0.74) and cognitive empathy (α = 0.67) subscales showed adequate internal consistency. Several prior studies have used the Greek version of the GEM in community samples of Greek Cypriot children (e.g., Georgiou et al., 2019a, 2019b; Kimonis et al., 2015). Moreover, we decided to use the GEM in the current study because it captures both subcomponents of empathy and not only an overall score of empathy.

### Autistic Traits

Autistic traits were measured using the school-age form of the Social Responsiveness Scale (SRS), a 65-item parent and/or teacher report (Constantino & Gruber, 2012). In the current study, SRS was used as a parent report. Parents rated their children on a 4-point Likert scale (0 = not true, 1 = sometimes true, 2 = often true, 3 = almost always true) with total scores ranging from 0 to 195, with higher scores indicating higher degrees of social impairment. SRS captures five domains/treatment subscales of autistic traits: awareness, cognition, communication, motivation, and mannerisms. In the current study, only the total score of SRS was used. Previous studies have verified that SRS shows high internal consistency (α = 0.91–0.97) and acceptable inter-rater reliability (0.76 and 0.95) (Bölte et al., 2008; Constantino & Todd, 2003; Constantino et al., 2000). In the current study, the total SRS score demonstrated excellent internal consistency (α = 0.92). SRS was used for measuring autistic traits since it captures a continuous dimensional distribution of autistic symptoms and not a categorization distribution using a cut-off score.

### Callous Unemotional Traits

CU traits were assessed with the parent-report version of the Inventory of Callous-Unemotional traits (ICU; Frick, 2004). ICU is a 24-items questionnaire, composed of 12 positively worded (e.g., “he/she express his/her feelings openly”) and 12 negatively worded items (e.g., “he/she does not feel remorseful when he/she does something wrong”). Parents rated their children on a four point Likert scale (0 = Not at all true, 1 = Somewhat true, 2 = Very true, 3 = Definitely true) with total scores ranging from 0 to 72. The ICU captures three dimensions of CU traits: callousness (e.g., “He/she does not care who he hurts to get what he wants”), unemotional (e.g., “He/she does not show his emotions to others”) and uncaring attitudes (e.g., reverse scored items: “He/she feels bad or guilty when he/she does something wrong”). In the current study, only the total score of ICU was used. Previous studies have verified that the total ICU scale shows acceptable internal consistency in different countries (e.g., Ezpeleta et al., 2013; Kimonis et al., 2015), and several studies have verified the validity of ICU in community samples of Greek Cypriot children (e.g., Fanti, 2013; Fanti et al., 2017). In the present study, the total ICU scale demonstrated good internal consistency (α = 0.88).

### Statistical Analysis

**Fig. 1:**
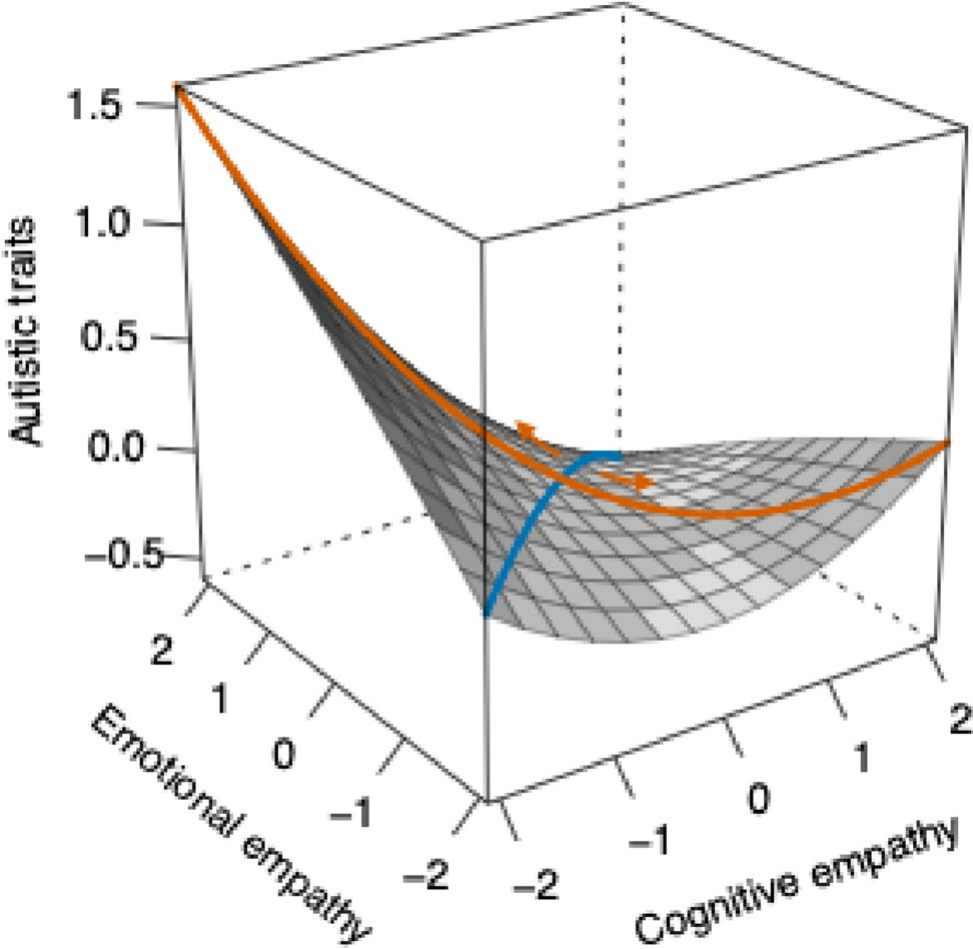
Polynomial regression with response surface analysis predicting autistic traits. *Note*. The orange line represents empathic disequilibrium, with movement from the center toward the left corner indicating emotional empathy dominance and movement toward the right corner indicating cognitive empathy dominance (see orange arrows). The blue line represents overall empathy, whereas movement along this line reflects increasing levels of overall empathy. The surface is presented in grayscale, with surface height reflecting standardized levels of autistic traits

All analyses were conducted using R v4.4.1 (R Core Team, 2021), using the *lavaan* package v.6–18 (Rosseel, 2012). Following the method previously outlined to capture empathic disequilibrium (Shalev et al., 2022, 2023), we conducted polynomial regression with response surface analysis (PRRSA; Edwards, 1994, 1995). This method allows to enter emotional and cognitive empathy into a single regression model following a polynomial equation (i.e., including cognitive and emotional empathy, their interaction, and their squared terms as predictors). The regression coefficients are then used to derive four surface parameters corresponding to both the linear and non-linear associations between the congruency (i.e., overall empathy) and incongruency (i.e., empathic disequilibrium) between emotional and cognitive empathy with the outcome. Linear associations with overall empathy and empathic disequilibrium are calculated by either adding or subtracting the coefficients of cognitive from emotional empathy, respectively. A negative score in the linear parameter of empathic disequilibrium indicates that emotional empathy exceeds cognitive empathy, and a positive value suggests the reverse. Non-linear parameters are calculated by adding or subtracting the interaction term coefficient from the combined effect of the squared terms of cognitive and emotional empathy (Edwards, 1994, 1995; Shanock et al., 2010). Simultaneously, a three-dimensional plot based on the regression coefficients is plotted, providing an informative way to assess overall empathy (blue line in Fig. 1) and empathic disequilibrium (black line in Fig. 1) while also gaining insights into cognitive and emotional empathy (Fig. 1). Such an approach provides a more reliable and interpretable method than traditional difference scores (Shanock et al., 2010).

Two main PRRSAs were conducted to predict autistic traits and callous-unemotional traits, controlling for the other outcome. Since empathy and its components are sensitive to age, sex, and gender (Rochat, 2023), we controlled for age and gender in both analyses.

Although PRRSA is preferred over difference scores, no validated method exists for using the PRRSA parameters as outcomes (Edwards, 1995). Therefore, to examine how age and gender predict empathic disequilibrium, we used difference scores (cognitive empathy minus emotional empathy), as was previously done in (Shalev et al., 2022; Shalev et al., 2025). Regression analysis was performed to examine how age, gender, and their interaction predict empathic disequilibrium, controlling for overall empathy (the sum of cognitive and emotional empathy).

## Results

The correlation matrix for the main variables is shown in Table 2. We first investigated how age and sex predicted empathic disequilibrium, controlling for overall empathy (see Table 3). Age was unrelated to empathic disequilibrium (*b* = -.25, 95% CI [-.54,.04], *p* =.09, *β* = -.20), nor were gender (*b* =.33, 95% CI [-.25,.91], *p* =.26, *β* =.09), or the interaction between gender and age (*b* = -.31, 95% CI [-.71,.09], *p* =.19, *β* = -.18). However, overall empathy was associated with empathic disequilibrium (*b* =.17, 95% CI [.02,.31], *p* =.02, *β* =.18). To further understand this association, a plot showing the correlation between empathic disequilibrium and total empathy is displayed in Supplementary Fig. 1.

**Table 2:** Correlation matrix

Variable	1	2	3	4	5
1. SRS					
2. ICU	.60***				
	[.47,.70]				
3. GEM—emotional empathy	-.09	-.23*			
	[-.26,.09]	[-.38, -.07]			
4. GEM—cognitive empathy	-.49***	-.46***	.07		
	[-.62, -.35]	[-.58, -.33]	[-.09,.23]		
5. Age	.28**	.22*	.03	-.04	
	[.11,.43]	[.06,.37]	[-.13,.20]	[-.21,.12]	
6. Gender	-.02	.03	-.07	.04	.10
	[-.19,.16]	[-.14,.19]	[-.23,.10]	[-.13,.20]	[-.07,.26]

*Note.* Values in square brackets indicate the 95% confidence interval for each correlation. Point-biserial correlations were used for gender. SRS – Social Responsiveness Scale; ICU – Inventory of Callous-Unemotional; GEM – Griffith Empathy Measure. * p <.05. ** p <.005***p < 0005

**Table 3:** Regression Predicting Empathic Disequilibrium

Predictor	*b*	95% CI	*β*	*p*
Age	−0.25	[−0.54, 0.04]	−0.20	.09
Gender	0.33	[−0.25, 0.91]	0.09	.26
Age × Gender	−0.31	[−0.71, 0.09]	−0.18	.19
Overall Empathy	0.17	[0.02, 0.31]	0.18	.02

### Predicting Autistic Traits

The polynomial regression model explained 45% of the variance in autistic traits. No gender differences were observed in autistic traits (*b* = −2.72, 95% CI [−8.33, 2.89], *p* =.34, *β* = -.06). However, age and CU traits were related to greater autistic traits (*b* = 24.79, 95% CI [16.58, 33.00], *p* = 3.25 × 10^–9^, *β* =.52). In line with our hypothesis, AE dominance was related to greater autistic traits (*b* = −43.12, 95% CI [−79.08, −7.16], *p* =.02, *β* = -.31) and overall empathy showed no linear (*b* = −19.44, 95% CI [−53.36, 14.48], *p* =.26, *β* = -.18), or non-linear (*b* = −54.07, 95% CI [−250.36, 142.22], *p* =.59, *β* = -.05) association with autistic traits. The non-linear association with empathic disequilibrium was also insignificant (*b* = 157.92, 95% CI [−116.00, 431.84], *p* =.26, *β* =.21). Figure 1 depicts the PRRSA plot, and polynomial regression estimates are detailed in Supplementary Table S1. The model residuals were approximately normally distributed, and the Variance Inflation Factor (VIF) for all variables ranged from 1.06 to 1.40, well below the threshold of 5, indicating no concern for substantial multilinearity (James, 2013).

### Predicting CU Traits

**Fig. 2:**
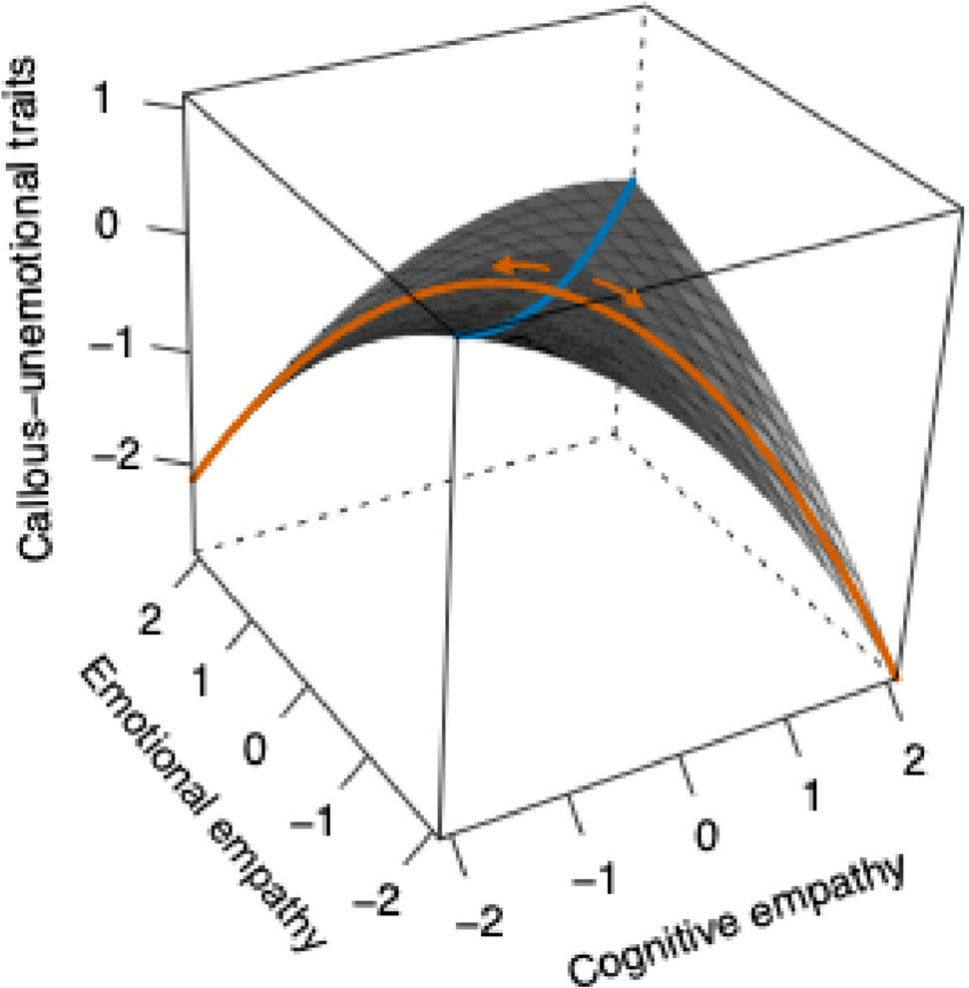
Polynomial regression with response surface analysis predicting callous-unemotional traits. *Note*. The orange line represents empathic disequilibrium, with movement from the center toward the left corner indicating emotional empathy dominance and movement toward the right corner indicating cognitive empathy dominance (see orange arrows). The blue line represents overall empathy, whereas movement along this line reflects increasing levels of overall empathy. The surface is presented in grayscale, with surface height reflecting standardized levels of callous-unemotional traits

The model accounted for 57% of the variance in callous-unemotional traits. No differences were found between boys and girls (*b* =.06, 95% CI [-.04,.16], *p* =.26, *β* =.07). However, older age (*b* =.04, 95% CI [.002,.08], *p* =.04, *β* =.13), and higher autistic traits (*b* =.01, 95% CI [.006,.01], *p* = 2 × 10^–9^, *β* =.41) were related to greater callous-unemotional traits. Empathic disequilibrium was non-linearly and negatively correlated with callous-unemotional traits (*b* = −9.15, 95% CI [−13.72, −4.57], *p* = 9 × 10^–5^, *β* = -.56) suggesting both forms of disequilibrium were related to lower callous-unemotional traits, with no linear association, suggesting no specific form is stronger than the other (*b* = -.08, 95% CI [-.56,.71], *p* =.81, *β* = -.06). Callous-unemotional traits were also predicted by lower overall empathy (*b* = −1.28, 95% CI [−1.84, −0.72], *p* = 8 × 10^–6^, *β* = -.40), with no non-linear association (*b* = 1.82, 95% CI [−1.75, 5.38], *p* =.32, *β* =.08). Residuals of the model were approximately normally distributed, with no substantial evidence of multicollinearity (VIF ranged from 1.06 to 1.45). A plot of the PRRSA is depicted in Fig. 2. The polynomial regression estimates are shown in Supplementary Table S1.

## Discussion

The current study aimed to explore the relationships among CU, autistic traits, and empathic disequilibrium in a sample of typically developing young children. Furthermore, it sought to investigate the effects of age and sex on empathic disequilibrium. This research aimed to address a notable gap in the existing literature, which primarily focused on examining the unique relationship between each trait with AE and CE separately, rather than the interplay between the two dimensions of empathy, specifically, empathic disequilibrium. The study's main findings indicated that AE dominance was related to a significant presence of autistic traits. In contrast, findings suggest that empathy imbalances — whether CE or AE dominance — were associated with lower CU traits. Before examining the key findings, all analyses (including correlation analysis and regression models) indicated a positive relation between high levels of CU traits and autistic traits. These findings may support the hypothesis of a subgroup within the population that exhibits co-occurrence between CU and autistic traits in children (for a review see (D. Murphy et al., 2024), with evidence supporting this idea for both clinical (Leno et al., 2015) and non-clinical samples (Pasalich et al., 2014). Specifically, Rogers et al. (2006) suggested that CU and autistic traits may co-occur in some populations, yet they do not form a single construct. This psychological condition is referred to as a “double-hit," representing a group of individuals exhibiting both traits and tend to demonstrate weak moral standards, engage in significant disruptive behaviors, and struggle with identifying facial expressions. While the current study did not explicitly investigate co-occurrence, the results support the proposal that empathic disequilibrium may serve as a transdiagnostic socio-emotional dimension (Shalev & Uzefovsky, 2025), suggesting further research into this concept could be valuable.

### Autistic Traits and Empathic Disequilibrium

The study supported our hypothesis that children with higher autistic traits are more likely to experience an empathic imbalance favouring AE. These findings align with earlier studies in adults (e.g., Shalev & Uzefovsky, 2020; Shalev et al., 2022, 2023), indicating that individuals with high levels of autistic traits may face an imbalance in their capacity to understand and experience others' emotions. Their ability to resonate with others' feelings appears to dominate their understanding of those emotions. Our findings demonstrate for the first time that this pattern is already evident in childhood. This is also consistent with the developmental lag of CE. As is well-documented, AE is evident in children's lives from an early age, even in infants. In contrast, CE develops later and relies on executive functioning and social learning (see (Uzefovsky & Knafo-Noam, 2016). Consequently, children with high levels of autistic traits may encounter both challenges and delays in theory of mind (ToM) development, resulting in this imbalance during early childhood. Our findings provide empirical support for the notion that empathic disequilibrium is a valuable predictor of autistic traits, reinforcing its critical role in understanding these traits. The absence of significant results for overall empathy scores is consistent with the notion that overall empathy is not as strong a predictor of autistic traits as empathic disequilibrium and aligns with previous findings (Moseley et al., 2024). This finding reinforces the view that theories should focus on the intrapersonal relationships among empathy subcomponents, rather than solely addressing a global or specific empathy deficit.

Regarding sex, the lack of differences suggests that the AE dominance profile is equally common in boys and girls. These findings reinforce previous research showing that, although empathic disequilibrium is strongly influenced by sex in adulthood, no differences are observed between boys and girls during childhood (Shalev et al., 2025). This suggests that sex-related differences may emerge later in development, possibly due to increased social expectations with the onset of school (around age 5) or biological changes during adolescence, which make these differences more noticeable (De Bolle et al., 2015). However, we did not examine the variables associated with age differences; hence, our conclusions cannot be distinctly articulated.

### CU Traits and Empathic Disequilibrium

Interestingly, our results revealed a negative association between CU traits and empathic disequilibrium, suggesting that both forms of empathic disequilibrium are linked to lower CU levels. This contrasts with previous longitudinal findings showing that children who later developed high CU traits tended to remain in a state of AE-dominance for a longer period and did not reach equilibrium (Shalev et al., 2025). Unlike the earlier study, which tracked changes over time, the current study is cross-sectional, providing a snapshot of the current state of the child’s empathic disequilibrium and CU traits, rather than their developmental patterns. Additionally, our sample had a higher average age (7.3 years), a stage at which, in the earlier study, many children were suggested to have already experienced both types of empathic disequilibrium. That prior experience of both disequilibrium tendencies during development was interpreted as a sign of adaptive and flexible use of different strategies for responding to others’ emotions. Therefore, although our findings contradict the initial hypothesis of greater AE-dominance in children with high CU traits, the observed tendency toward lower levels of both AE- and CE-dominance may reflect a lack of flexibility in navigating empathic responses during development. Interestingly, a similar, yet non-significant, trend was also observed in adults with high psychopathic traits (Shalev et al., 2023). This highlights the potential adaptive function of empathic disequilibrium during development, and contrasts children with high CU traits from those with high autistic traits.

### Strengths, Limitations, and Conclusions

The current research has notable strengths and weaknesses worth acknowledging. Among its strengths is the use of questionnaires specifically designed for younger children. Additionally, consistent with previous studies (e.g., Kimonis et al., 2015), both high-risk and control groups were included to enhance variability in CU and autistic traits. Regarding limitations, a review by Murphy (2019) suggested that the GEM AE scale primarily measures emotional contagion rather than comprehensive affective sharing, while the CE scale is primarily linked to cold-heartedness or callousness, potentially lacking clarity as a measure of empathy. However, Dadds et al., (2008) argued that GEM has strong empirical backing, citing factor analysis across age and gender, inter-rater reliability, and the theoretical soundness of assessing the cognitive aspects of empathy. Additionally, the empathy assessment relied entirely on parental reports, reflecting parents’ perceptions of their child’s empathic skills. Although parents play a critical role in reporting children’s behaviors, emotions, and competencies (De Los Reyes & Kazdin, 2005), future studies could benefit from incorporating multi-informant assessments that include input from parents, teachers, child self-reports, and physiological responses during empathy-eliciting tasks. Moreover, because parents completed all measures in the present study, shared-method variance may have inflated the observed associations between empathy, CU traits, and autistic traits. To minimize the influence of common method bias, future research should integrate multi-informant approaches (e.g., teacher reports, clinician ratings) and task-based assessments (Podsakoff et al., 2003). Furthermore, while several participants in our study displayed increased CU and autistic traits, we excluded individuals who fulfilled the clinical criteria for autism or the ‘limited prosocial emotions’ specifier of conduct disorders as outlined by the DSM-5. Therefore, upcoming research might benefit from utilizing a clinical sample to evaluate the generalizability of the findings across community and clinical populations. A further limitation of the current study is its reliance on total scores for both the ICU and the SRS, which may obscure specific associations between other trait dimensions and empathy. For ICU, these include callousness, uncaring, and emotional traits; and for SRS social communication/interaction difficulties, as well as restricted or repetitive behaviors and interests, as outlined by Frazier and colleagues (2014). Future research with higher statistical power could improve on this by exploring empathy in relation to empirically derived ICU facets and validated SRS factor structures, potentially providing a clearer and more accurate depiction.

Given these limitations, our findings build upon and expand prior research into the empathy profiles linked to higher CU and autistic traits in children. Our results indicate that children with high levels of CU traits tend to display lower levels of empathic disequilibrium; however, children with autistic traits demonstrate an empathic imbalance favouring AE, which is in line with the patterns found in adults. The findings related to CU traits provide a new understanding of these traits in early development, supporting the idea that earlier experience with both forms of empathic disequilibrium during development may be important for adaptive empathic reaction later on. Additionally, the overall low levels of total empathy suggest that children with high CU traits exhibit difficulties with both understanding and sharing emotions. Future research should focus on exploring how the interaction of CU and autistic traits affects empathic disequilibrium, which is essential for advancing our understanding of unique empathy profiles within these groups. This can enhance the effectiveness of current prevention and intervention programs and guide the development of new strategies that address the fundamental differences between CU traits, autistic traits, and their interaction. Moreover, considering Bird and Cook's (2013) proposal that many emotional difficulties traditionally linked to autism are better explained by alexithymia, controlling for alexithymia as a covariate will be crucial for future research to determine whether the empathic imbalance favoring AE is directly related to autistic traits or whether alexithymia functions as a potential moderator factor.

## Supplementary Information

Below is the link to the electronic supplementary material.

Supplementary file1 (DOCX 107 KB)
