# Supplementary material for: Empathic Disequilibrium in Autistic Traits and CU Traits: Investigating Empathy Imbalance in Children
Source: Res Child Adolesc Psychopathol. 2026 Feb 6;54(1):26. doi: 10.1007/s10802-025-01405-y (PMC12881055; doi:10.1007/s10802-025-01405-y)
Supplement: Supplementary file 1 — Supplementary file1 (DOCX 107 KB) [file 10802_2025_1405_MOESM1_ESM.docx]

**Supplementary Figure 1**

***Correlation between empathic disequilibrium and overall empathy***


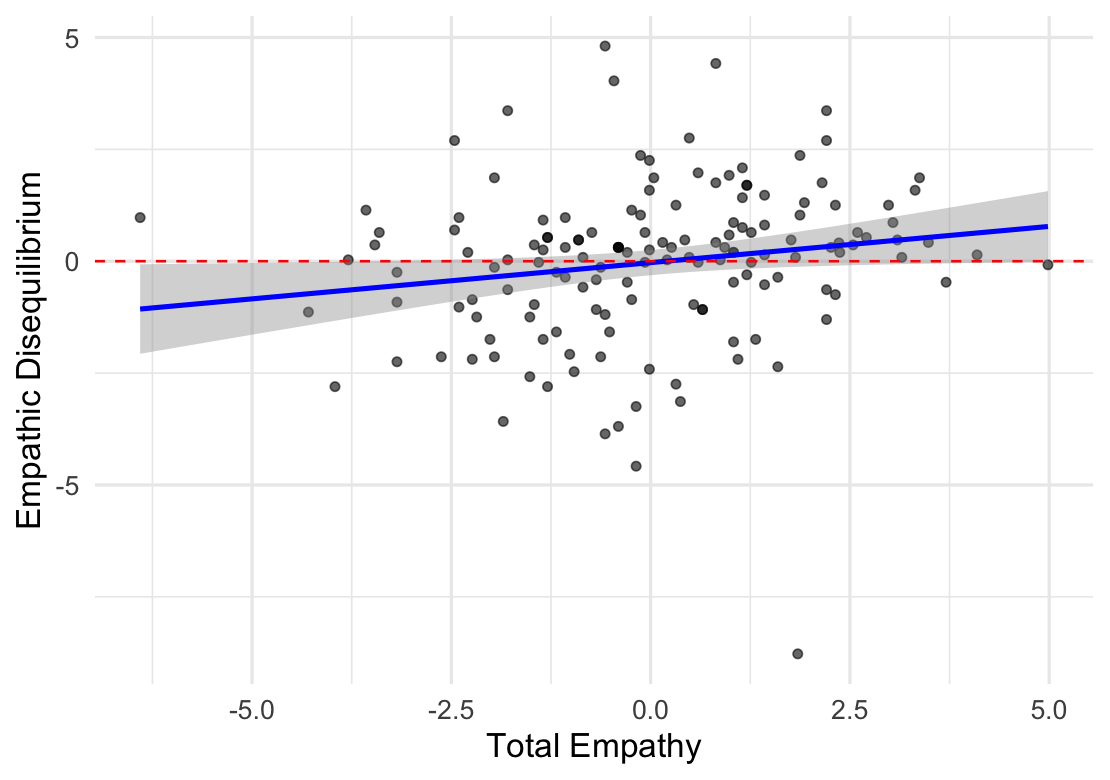


*Note*. The Red dashed line marks the point where emotional and cognitive empathy are equivalent (i.e., empathic disequilibrium equals 0).

**Supplementary Table S1**

|  | **CE** | **EE** | **CE2** | **CE * EE** | **EE2** |
| --- | --- | --- | --- | --- | --- |
| **Autistic traits** | **-31.28 [-50.71, -11.86]**** | 11.84 [-17.22, 40.90] | 60.95 [-40.06, 161.96] | -105.99 [-256.28, 44.29] | -9.03 [-190.24, 172.18] |
| **Callous-unemotional traits** | **-.60 [-.95, -.26]**** | **-.68 [-1.17, -.19]*** | **-2.09 [-3.87, -.32]*** | **5.48 [2.97, 8.00]***** | -1.57 [-4.80, 1.66] |

*Note*. * *p* < 0.05, ** *p* < 0.005, *** *p* < 0.0005
